# Successful surgical management of ventricular septal perforation following left ventricular free wall rupture due to acute myocardial infarction: a case report

**DOI:** 10.1186/s44215-024-00128-8

**Published:** 2024-02-22

**Authors:** Makoto Takehara, Sanae Tomotsuka, Shinichi Tsumaru, Takeshi Shimamoto

**Affiliations:** Department of Cardiovascular Surgery, Hamamatsu Rousai Hospital, 25 Shogen-Cho, Higashi-Ku, Hamamatsu-Shi, Shizuoka-Ken 430-8525 Japan

**Keywords:** Left ventricular free wall rupture, Ventricular septal perforation, Acute myocardial infarction, Mechanical complications

## Abstract

**Background:**

The mechanical complications associated with acute myocardial infarction can be fatal. We report a life-saving case of a very elderly patient with consecutive mechanical complications of acute myocardial infarction.

**Case presentation:**

A 90-year-old woman was transferred to our hospital for emergency care due to chest pain. She underwent emergency percutaneous coronary intervention of the left anterior descending branch and was diagnosed with acute myocardial infarction and intra-aortic balloon pumping insertion was performed. After returning to the intensive care unit, she experienced chest pain and went into shock. Echocardiography showed left ventricular free wall rupture, and percutaneous cardiopulmonary support was established. Emergency left ventriculoplasty was performed for the left ventricular free wall rupture. A ventricular septal perforation was observed on postoperative day 9, and its closure through right ventriculotomy was performed on postoperative day 10. On the 128th postoperative day, the patient was transferred to the hospital for rehabilitation.

**Conclusions:**

A 90-year-old patient with consecutive left ventricular free wall rupture and ventricular septal perforation after acute myocardial infarction was successfully managed with two surgeries.

## Background

Left ventricular free wall rupture (LVFWR) occurs in 1–4% of patients after acute myocardial infarction (AMI), but is reported to account for 10–20% of patient deaths [1, 2]. Ventricular septal perforation (VSP) occurs in 1–2% of AMI cases, with an in-hospital mortality rate of approximately 45% for surgically treated patients and 90% for medically treated ones, resulting in a very poor prognosis [3, 4]. Herein, we describe a life-saving case of consecutive LVFWR and VSP after AMI in a 90-year-old woman who underwent two emergency surgeries.

## Case presentation

A 90-year-old woman was referred to our hospital for emergency care for chest pain. She had no significant past medical history. Her activities of daily living were good and she had no cognitive problems. The patient was 147 cm tall, weighed 37 kg, and had a BMI of 17 kg/m^2^. Preoperative standard 12-lead electrocardiography showed complete right bundle branch block and ST-segment elevation in leads V1, V2, V3, V4, V5, I, and aVL, and depression in leads II, III, and aVF. The serum creatine kinase level (81 U/L) was not elevated. Emergent coronary angiography revealed total occlusion of the proximal left anterior descending branch (LAD), and she underwent emergency percutaneous coronary intervention (PCI) of the LAD with intra-aortic balloon pumping (IABP) insertion. An hour after the PCI, the patient suddenly experienced shock. Echocardiography revealed massive pericardial effusion and a subepicardial hematoma of the anterior wall. After inserting percutaneous cardiopulmonary support (PCPS), we performed emergency surgery with a diagnosis of cardiac tamponade caused by LVFWR.

**Fig. 1:**
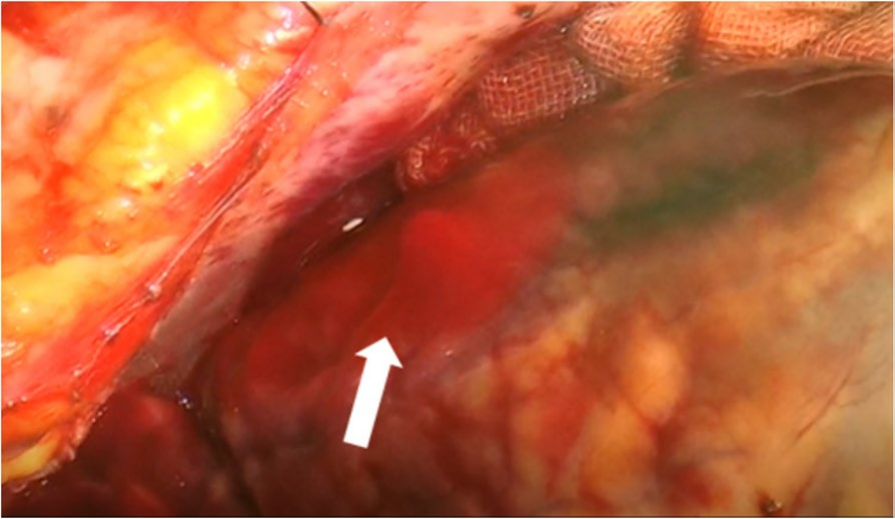
Intraoperative findings of left ventricular free wall rupture-1. Pulsatile bleeding and myocardial infarction in the anterior left ventricular(the white arrow)

**Fig. 2:**
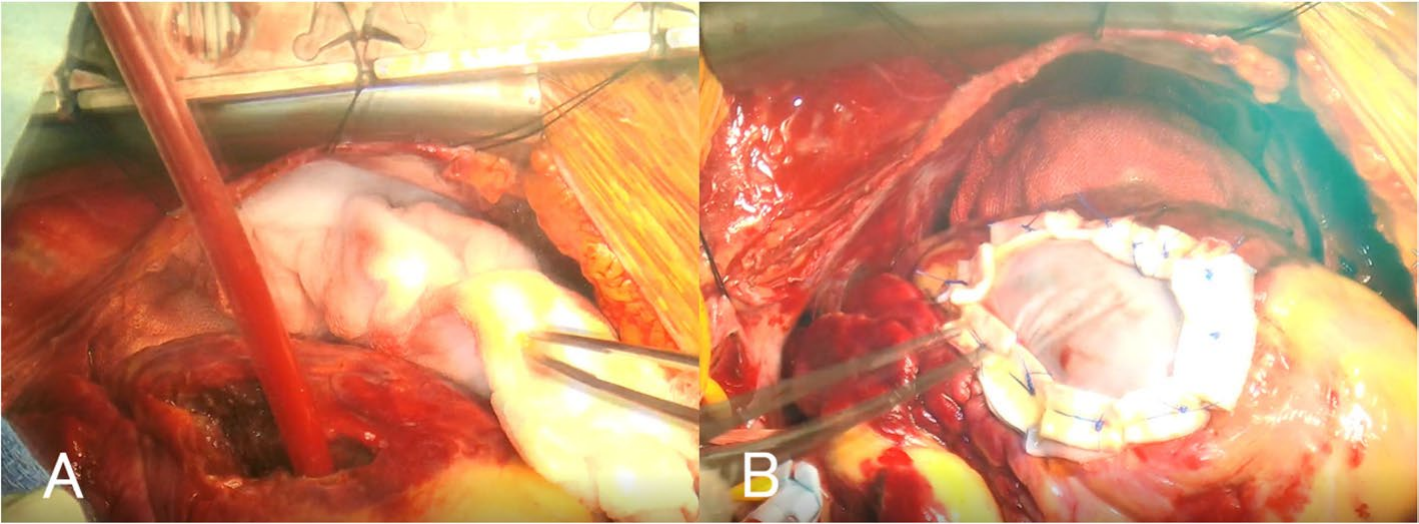
Intraoperative findings of left ventricular free wall rupture-2. **A** Fragile tissues around the rupture are trimmed. **B** Left ventricular free wall rupture closed with bovine pericardial patches

After pericardiotomy, bloody pericardial effusion and pulsatile bleeding were observed in the LAD lesion (Fig. 1). The patient was diagnosed with blow-out type LVFWR. Total cardiopulmonary bypass (CPB) was performed via cannulation of the ascending aorta and direct bicaval cannulation. Myocardial protection was achieved by cardioplegia delivered in an antegrade manner through the ascending aorta. Ventriculotomy was performed through an incision extending from the perforation parallel to the LAD. Myocardial necrotic tissue was removed to the fullest extent possible (Fig. 2A). Considering the flexibility of the left ventricle (LV), bovine pericardial patch was selected for emergency surgery. The patch was cut into two 7 cm × 5 cm pieces and was placed over the rupture hole from the endocardial side. Left ventriculoplasty was successfully performed with two patches placed inside and outside the rupture hole in a sandwich manner (Fig. 2B). Eleven horizontal mattress sutures (3–0 polypropylene) were placed transmurally from the endocardial to the epicardial side. The patch and the felts on the epicardial patch were securely tied. BioGlue (CryoLife, Inc. Kennesaw, GA, USA) was applied between the patches before tying all sutures. She was weaned from CPB; however, the operation was completed with her chest remaining open because of hypotension. The operative duration was 264 min. Aortic clamping took 72 min. The chest was closed 1 day after the surgery. IABP was discontinued 7 days postoperatively.

**Fig. 3:**
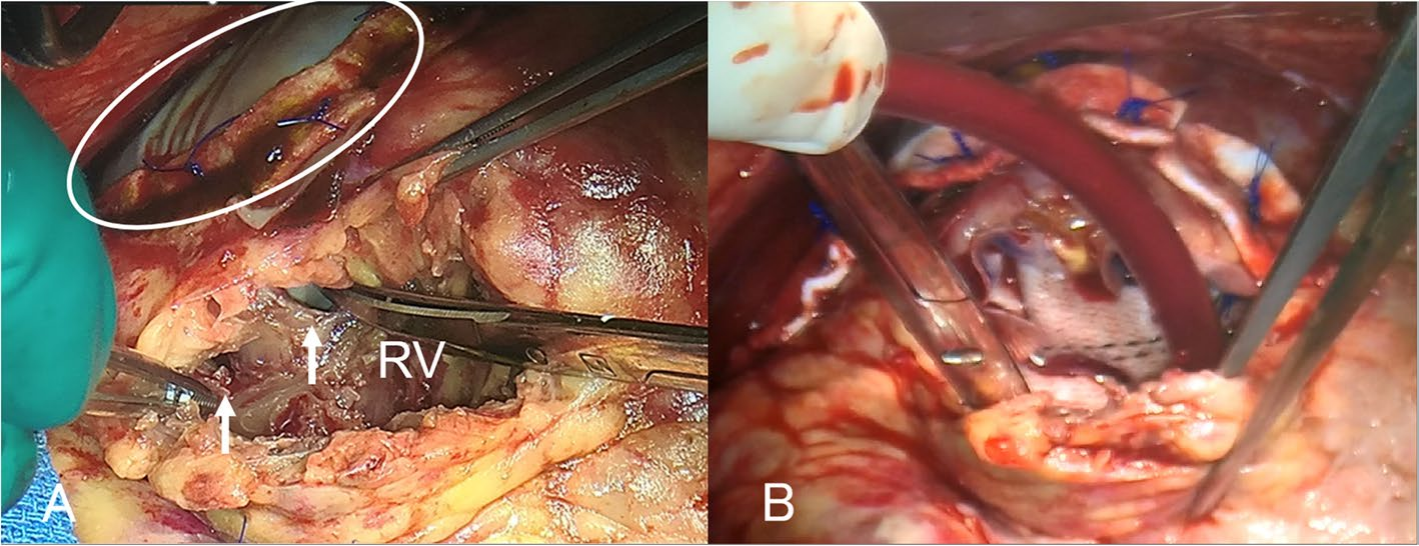
Intraoperative findings of ventricular septal perforation. **A** The white arrows indicate ventricular septal perforation. The circle line indicates free wall patch was repaired at the first surgery. **B** Ventricular septal perforation closed with Hemashield patches ((Maquet GmbH & Co KG, Rastatt, Germany)

**Fig. 4:**
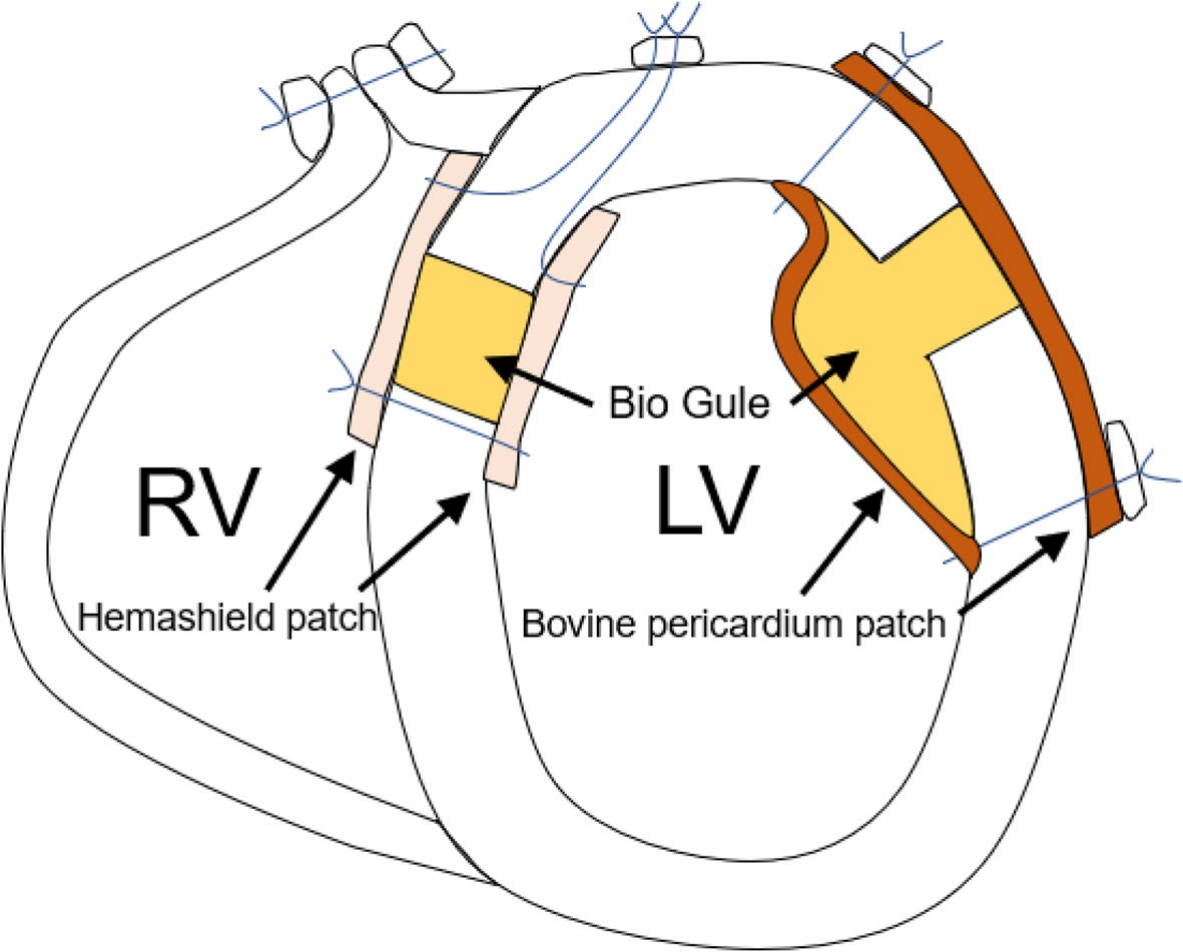
Schematic representation of the first and the second surgery. Sandwich repair technique via the right ventricular approach and left ventriculoplasty with patch closure were performed

However, 9 days after the initial surgery, a systolic murmur was detected. Color-Doppler echocardiography revealed two shunt flows across the ventricular septum. The pulmonary-to-systemic blood flow ratio was 2.4. The patient was diagnosed with VSP after AMI. On the 10th postoperative day, a second surgery was performed for the VSP. A repeat midline sternotomy was performed. Total CPB was established in a manner similar to that of the initial operation. An incision of the right ventricle (RV) parallel to the LAD revealed the VSP and myocardial necrotic tissue at the apex (Fig. 3A). A Hemashield patch (Maquet GmbH & Co., KG, Rastatt, Germany) was selected considering its usability. Two pieces of Hemashield patches with the size of 7 cm × 5 cm in size, were prepared. Four 3–0 Prolene mattress sutures were placed over the LV side patch transmurally from the LV cavity to the LV free wall. Another four 3–0 Prolene mattress sutures were placed from the LV side patch to the RV and the RV side patch. Four 3–0 Prolene mattress sutures were placed over the RV side patch transmurally to the LV free wall. BioGlue was applied between the Hemashield patches before knotting all sutures. VSP was closed using the extended sandwich technique via the RV approach (Fig. 3B), and the right ventriculotomy was closed with a horizontal mattress and over-and-over sutures, buttressed with Teflon felt strips. Figure 4 shows a schematic representation of the sandwich repair technique using the RV approach and left ventriculoplasty with patch closure. Surgery was completed with the chest remaining open because of hypotension caused by chest closure. The operative duration was 275 min. The aortic clamping took 91 min. The chest was closed on postoperative day 3.

Postoperative echocardiography revealed an LV end-diastolic diameter of 38 mm, LV ejection fraction of 48%, no residual shunt. It was possible to withdraw the patient from the IABP 18 days after the second surgery. Subsequently, her postoperative course was uneventful. On the 118th postoperative day, the patient was discharged on her feet without assistance from a cane to another hospital for further rehabilitation.

## Discussion

There have been some case reports of double cardiac complications in myocardial infarction with simultaneous VSP and LVFWR [5]. However, to the best of our knowledge, only two case reports of VSP after LVFWR treatment have been published [6, 7]. VSP and LVFWR rarely occur consecutively in the same patient. Due to therapeutic advances such as PCI and thrombolytics, the prevalence of mechanical complications due to myocardial infarction appears to have decreased, but hospital mortality is still high in patients with LVFWR (80%) or VSP (41%) [8]. In the Society of Thoracic Surgeons Adult Cardiac Surgery Database, 2876 patients with post-MI VSP underwent surgical repair from 1999 to 2010, and a high operative mortality rate of 42.9% was observed in the overall cohort, which was the highest mortality of all cardiac surgery among the patients registered in this database [9]. A shorter interval between the onset of these complications and the subsequent operation is a predictor of outcome regardless of the patient’s preoperative condition [10]. LVFWRs are clinically classified as oozing or blow-out types [11]. The sutureless technique with a surgical adhesive is commonly used in the treatment of the oozing type because it is simple and effective without the need for sutures on the fragile myocardium [12]. Perfect hemostasis is technically demanding in the blowout type. Sutureless techniques are usually unable to achieve hemostasis, and direct or patch closure is the procedure of choice [12, 13]. The suture lines of direct closure and patch closure must be along the nonischemic myocardium, and transmural stitches are necessary, which sometimes reduce the left ventricular function because of the sutures on the nonischemic myocardium [14]. In our case, a blowout-type LVFWR was diagnosed in the anterior aspect of the LV near the diagonal branch. The sutureless technique was not applicable for hemostasis because the area of the necrotic myocardium was large, and bovine pericardial patch closure was performed.

For the treatment of VSP, we performed a sandwich technique via an RV. This technique is useful in the treatment of VSP with two large patches that sandwich the VSP and the surgical adhesive for the fixation of the patches, which might have advantages because of less suture tension so as not to tear the myocardium. Hemostasis through a right ventriculotomy is relatively easy to achieve. Moreover, if we performed repair through left ventriculotomy, an additional LV incision with possible bleeding between the two repairs could have occurred, which is a surgical disaster. VSP repair through right ventriculotomy is simpler than the infarct exclusion technique because surgeons do not change the left ventricular morphology with sutures and patches [15]. Kinoshita et al. reported that the extended sandwich patch technique can safely and easily eliminate leakage without loss of RV function, even in the acute phase [16]. In our case, the LV anterior wall was patched, and if an LV approach had been used, it would have been necessary to remove the LV anterior wall patch. Therefore, an RV approach was used.

## Conclusions

A 90-year-old patient with consecutive left ventricular free wall rupture and ventricular septal perforation after acute myocardial infarction was successfully managed with two surgeries.

## Data Availability

The datasets used and/or analyzed during the current study are available from the corresponding author on reasonable request.

## Abbreviations

LVFWR: Left ventricular free wall rupture
AMI: Acute myocardial infarction
VSP: Ventricular septal perforation
LAD: Left anterior descending branch
PCI: Percutaneous coronary intervention
IABP: Intra-aortic balloon pumping
PCPS: Percutaneous cardiopulmonary support
CPB: Cardiopulmonary bypass
LV: Left ventricle
RV: Right ventricle
